# Children and adolescents living with human immunodeficiency virus/acquired immunodeficiency syndrome in a tertiary hospital in southern Brazil

**DOI:** 10.1590/1984-0462/2025/43/2025006

**Published:** 2025-12-01

**Authors:** Savas Sobral Silveira, Marina Pinheiro da Silva Bolinsenha, Yasmin Oliveira Rossoni, Sinis Sobral Silveira, Giuliana Lugarini, Tony Tannous Tahan, Tatiane Emi Hirose, Cristina de Oliveira Rodrigues, Lucca Weffort Caprilhone, Andrea Maciel de Oliveira Rossoni, Betina Mendez Alcântara Gabardo

**Affiliations:** a Universidade Federal do Paraná, Curitiba, PR, Brazil.; b UniversidadePositivo, Curitiba, PR, Brazil.

**Keywords:** HIV, Children, Viral load, Clinical classification, Immunological classification, HIV, Criança, Carga viral, Classificação clínica, Classificação imunológica

## Abstract

**Objective:**

The aim of the study was to describe the clinical and epidemiological features of pediatric patients living with human immunodeficiency virus in a tertiary care service in southern Brazil and the types of antiretroviral therapy (ART) used, and to compare the immunological and virological profiles at diagnosis and at the present time.

**Methods:**

This longitudinal, observational study involved retrospective data collection, evaluating the clinical, immunological, and virological profiles of patients treated at a pediatric infectious disease outpatient clinic in a tertiary care center in Paraná during 2022.

**Results:**

A total of 52 patients were included. Median age at diagnosis was 4.0 years; current median age, 10.5 years; 55.7% were girls. Most (86.5%) were cared for by parents or extended family, and 9.6% were on a second-line ART regimen. Neuropsychiatric alterations occurred in 26.9%; dyslipidemia in 66.0%. The frequency of immunosuppressed patients decreased from 35.3% to 24.4%, and the frequency of clinical symptoms from 11.6 to 1.9% (both p<0.05). The mean viral load (log) dropped from 4.62 to 3.01. No statistically significant associations were found between age, duration of follow-up, or regularity of follow-up and clinical changes.

**Conclusions:**

The use of ART effectively reduced viral load, symptoms, and immunosuppression. Despite improvements, a considerable percentage still shows positive viral load, indicating risk of future complications.

## RESUMO

**Objetivo:**

Descrever as características clínicas e epidemiológicas, bem como os tipos de terapia antirretroviral (TARV) utilizados, e comparar o perfil imunológico e virológico de pacientes pediátricos vivendo com HIV em um serviço de atenção terciária no sul do Brasil, no momento do diagnóstico e no momento atual.

**Métodos:**

Estudo observacional, longitudinal, com coleta de dados retrospectiva, avaliando-se perfil clínico, imunológico e virológico, com inclusão dos pacientes atendidos no ambulatório de infectologia pediátrica em centro de atenção terciária no Paraná, no ano de 2022.

**Resultados:**

Participaram do estudo 52 pacientes. A mediana de idade ao diagnóstico foi de 4,0 anos (0 a 10) e a atual, de 10,5 anos (1 a 17); 55,7% eram meninas; 86,5% eram tuteladas pelos pais ou família extensa; 9,6% dos pacientes estavam em uso de esquema de TARV de falência. As principais alterações encontradas foram neuropsiquiátricas (26,9%) e dislipidemia (66,0%). A frequência de imunossuprimidos passou de 35,3% para 24,4% e a frequência de pacientes com sintomas clínicos passou de 11,6 para 1,9% (ambos p<0,05). Além disso, a carga viral média (log) baixou de 4,62 cópias para 3,01. Não foi encontrada relação estatisticamente significativa entre idade, tempo de acompanhamento ou regularidade de acompanhamento e melhora ou piora do paciente.

**Conclusões:**

O uso de TARV reduziu a carga viral, a sintomatologia e a imunossupressão. Mesmo com as melhoras, uma porcentagem ainda alta desses indivíduos permanece com carga viral positiva, o que pode ser o primeiro passo para complicações futuras.

## INTRODUCTION

Infection with the human immunodeficiency virus (HIV) remains a global public health problem. Among the many facets of the HIV issue, one is the infection of children and adolescents. This infection is transmitted horizontally (through sexual or parenteral routes) or vertically (from an HIV-positive mother transmitting the virus to her newborn). The primary source of transmission is vertical, and despite measures aimed at its eradication, approximately 180,000 children continue to be infected worldwide each year, with around 1.8 million living with the virus.^1^ These rates are a direct consequence of 15% of the total number of pregnant women living with HIV worldwide not having access to antiretroviral therapy (ART) during pregnancy.^2^ Even with improvements in the prevention of vertical transmission, which decreased from a peak of 43.1 per 100,000 live births in 1992 to 0.8 per 100,000 live births in 2019,^3^ the current prevalence still indicates that children continue to be affected by HIV. In addition to the intrinsic social and cognitive vulnerabilities of this population, there is also a biological aspect. The pediatric population experienced a global mortality rate of approximately 110,000 deaths in 2017, largely due to only 48% having access to ART.^1^

In cases of infection in children, the initial clinical manifestations are often nonspecific, including lymphadenopathy, hepatosplenomegaly, recurrent oral candidiasis, delays in neuropsychomotor development, and fluctuations or maintenance of weight and height curves. On the other hand, late clinical manifestations also include severe recurrent infections or opportunistic infections (primarily pneumonia caused by *Pneumocystis jirovecii*).^4^ This necessitates a high degree of suspicion to consider the diagnosis, leading to delays in confirming the condition and initiating treatment.

According to current protocols, the initiation of treatment immediately after diagnosis is ideally recommended for any child diagnosed with HIV, regardless of the mode of transmission or the presence of symptoms.^3,5,6^ The earlier the initiation of ART, the lower the viral replication in the bloodstream and in reservoirs, resulting in reduced chronic inflammation and fewer complications.^7,8^

The availability of effective treatments today has transformed HIV into a chronic disease due to improved survival rates. In Brazil, access to ART has altered the patient profile, resulting in fewer children being born with the virus and more adolescents reaching adulthood.^9^ This improvement brings new challenges, such as reducing loss to follow-up, maintaining medication adherence, and managing the long-term effects of ART.^9^ For this reason, this study aimed at describing the clinical and epidemiological features of pediatric patients living with HIV and comparing the immunological and virological profile at diagnosis and at the present time in a tertiary care service in southern Brazil.

## METHOD

This was a retrospective, longitudinal, analytical, and observational study. All children and adolescents living with human immunodeficiency virus/acquired immunodeficiency syndrome (HIV/AIDS) who attended the outpatient clinic for secondary immunodeficiency at the Pediatric Infectious Diseases Service of the Clinical Hospital of Federal University of Paraná (CH-UFPR) during the year 2022 were included. Patients without a documented medical history were excluded. Data collection occurred in July and August of 2023. Informed Consent Forms (TCLE) and Assent Forms (TALE) were applied to the patients, as required by the Ethics Committee for Research Involving Human Beings at CH-UFPR. The present research was approved with the identification number 69036123.9.0000.0096.

Among the data collected from each patient’s medical record were: sex, age, weight, and height at the last visit, legal guardians of the minor, origin, mode of virus transmission (breastfeeding transmission was considered when the mother had a negative rapid test at delivery with a subsequent HIV diagnosis), comorbidities, number of changes in ART regimen, current regimen, presence or absence of HIV genotyping (if genotyping was present, whether it was primary and if there was any description of resistance), viral subtype, use of prophylaxis for opportunistic infections, first and last viral load (the minimum limit of detection being 20 copies/mL and maximum 10,000,000 copies/mL), first and last Cluster of Differentiation 4 (CD4) count (immunological classification), presence of other associated symptoms initially and at the last visit (clinical classification), presence of alterations in laboratory tests (renal function, liver function, complete blood count, and lipid profile) at the last visit, results of serologies (toxoplasmosis, syphilis, Cytomegalovirus, Epstein-Barr virus, hepatitis A, hepatitis B, hepatitis C, and Human T-lymphotropic virus), and adherence to the vaccination schedule specific to this population. The regularity of attendance at consultations was also assessed, considering a patient irregular if they had more than two consecutive absences during the period. Additionally, it was analyzed whether the patient showed improvement or not in clinical, immunological, and virological aspects, with improvement defined as a change in clinical classification (reduction of associated symptoms), an increase in CD4 levels, or an undetectable viral load.

Height and weight percentiles were defined according to the World Health Organization growth curves. For laboratory tests, standardized criteria from the Diagnostic Support Unit of CHC-UFPR were used. The immunological and clinical classification of the children followed the guidelines proposed in the Clinical Protocol and Therapeutic Guidelines for Managing HIV Infection in Children and Adolescents by the Ministry of Health in the version in force at the time of data collection.^5,6^

Once collected, the data were organized into a Microsoft Excel^®^ spreadsheet and analyzed using Stata/SE 12.0^®^. The variables were described using frequency counts, means, or medians. Viral and immunological statuses were compared between treatment start and current follow-up using the McNemar test. Statistical significance was considered at p<0.05.

## RESULTS

During the study period, 53 patients were treated in the service. One patient was excluded because they were a foreign child who had recently arrived in Brazil, without any previous history of tests or treatment available. This resulted in a total sample of 52 patients.

**Figure 1:**
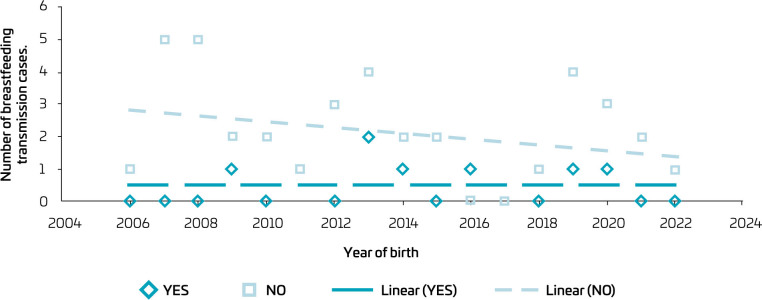
Number of cases of vertical transmission through breastfeeding by year of birth in children and adolescents living with HIV/AIDS, treated at a referral service in southern Brazil, 2022.

Data on gender, age, residency, legal guardianship, age of diagnosis, and form of transmission are shown in Table 1. Regarding the forms of vertical transmission, Figure 1 shows that all forms of vertical transmission have been decreasing except for transmission through breastfeeding. Comorbidities, coinfections, and laboratory abnormalities found are listed in Table 2.

**Table 1:** Epidemiological characteristics of children and adolescents living with human immunodeficiency virus, treated at a referral service in southern Brazil, 2022.

	n	%
Gender		
Female	29	55.7
Male	23	44.3
Age (years old)		
>0 and ≤5	13	25.0
>5 and ≤10	13	25.0
>10 and ≤17	26	50.0
Residency		
Curitiba	24	46.1
Metropolitan Region of Curitiba	20	38.5
Other State of Paraná cities	8	15.4
Legal guardianship		
Biological parents	29	55.8
Other biological relatives	16	30.7
Foster parents	7	13.5
Transmission mode		
Vertical	51	98.0
Intrauterine	43	84.4
Breastfeeding	8	15.6
Unknown	1	2.0
Age of diagnosis (median 4 years old)		
>0 and ≤5	29	55.7
>5 and ≤10	23	44.3

**Table 2:** Comorbidities, laboratory abnormalities, and coinfections of children and adolescents living with human immunodeficiency virus, treated at a referral service in southern Brazil, 2022.

	n	%
Comorbidities		
Neuropsychiatric	14	26.9
Skin	7	13.5
Respiratory tract	4	7.7
Liver	4	7.7
Genitourinary tract	2	3.9
Endocrine	2	3.9
Cardiovascular	1	1.9
Laboratory abnormalities		
Lipid profile	17	66.0
Microalbuminuria	3	5.7
Liver function	2	3.8
Thrombocytopenia	2	3.8
Anemia	1	1.9
Thrombocytosis	1	1.9
Coinfections		
Immune to CMV	38	73.1
Immune to EBV	29	55.7
Immune to toxoplasmosis	12	23.1
Congenital syphilis	1	1.9
Congenital CMV	1	1.9
Hepatitis B	1	1.9

CMV: Cytomegalovirus; EBV: Epstein-Barr vírus.

Among the more severe complications, 1 (1.9%) child presented with congenital CMV infection, characterized by pancytopenia and significant neurological impairment, 1 (1.9%) had miliary tuberculosis, and 1 (1.9%) had pneumocystosis. One (1.9%) child died at the age of 4 due to a case of fulminant hepatitis of undetermined cause, requiring a liver transplant.

Regarding the use of specific prophylaxes, only 5 (9.6%) were receiving prophylaxis for *P. jirovecii,* and 1 (1.9%) was on prophylaxis for atypical mycobacterial infections. No patients were on prophylaxis for tuberculosis, herpes, fungi, or toxoplasmosis. In clinical follow-up, it was found that 32 (61.5%) underwent screening for latent tuberculosis infection, and among those, 6 (18.7%) were infected and had already completed appropriate treatment for latent infection. Concerning cardiac alterations, 33 (63.4%) had an echocardiogram, and among those, 2 (6%) had a patent foramen ovale (PFO), 1 (3%) had an atrial septal defect (ASD), and 1 (3%) had tricuspid regurgitation. Ophthalmological evaluation was performed on 30 (57.9%) patients, of whom 1 (3.3%) showed an alteration in the fundoscopic exam. As for the nutritional status of the patients, their mean body mass index was 17.93 (±3.24), with a mean Z score of -0.71 (±1.38); 40 (76.9%) were classified as eutrophic, 6 (11.5%) were classified as underweight, 4 (7.7%) were at risk of overweight, 1 (1.9%) was classified as overweight, and 1 (1.9%) was classified as severely underweight.

Children and adolescents living with HIV/AIDS showed a statistically significant improvement when comparing the start of follow-up to the last evaluation regarding clinical, immunological, and virological classification (Table 3).

**Table 3:** Comparison of clinical-immunological classification and viral load measurements at the start of follow-up and the last evaluation of children and adolescents living with human immunodeficiency virus, treated at a referral service in southern Brazil, 2022.

Parameter	Initial		Current	
	n	%	n	%
Immunological classification (immunosuppression)*				
No	33	64.7	44	84.6
Moderate	12	23.5	7	13.5
Severe	6	11.8	1	1.9
Clinical classification^†^				
A	1	1.9	0	0
B	4	7.7	0	0
C	2	3.9	1	1.9
N	45	86.5	51	98.1
Viral load (copies/mL)				
<20	15	28.9	33	63.5
20–1000	6	11.5	10	19.2
>1000	31	59.6	9	17.3
Value – log				
Minimum	0 (log 1.30)		0 (log 1.30)	
Maximum	10,000,000 (log 7.00)		413,143 (log 5.61)	
Mean	504,138 (log 4.62)		9,738 (log 3.01)	
95%CI (value)	65,522–942,754		-6,229 to 25,707	

p<0.05.

*No immunosuppression versus moderate or severe immunosuppression

^†^A or N versus B or C.

In the monitored population, 20 (38.5%) children or adolescents did not achieve an undetectable viral load. The average age of this group was 7.4 years [±0.99; 95% confidence interval (CI) 5.388–9.512], and the average follow-up duration was 5.15 years (±0.51; 95%CI 4.073–6.227).

When comparing the 44 patients who showed clinical/immunological/virological improvement to the eight who did not, there was no difference in follow-up duration (6.6 years, ±0.40; 95%CI 5.8–7.4 vs. 6.5 years, ±0.92; 95%CI 4.3–8.6, respectively) or in the current age of the children and adolescents (10.2 years, ±0.73; 95%CI 8.7–11.7 vs. 10.1 years, ±1.6; 95%CI 6.2–14.0, respectively).

Regarding improvement, the regularity of attendance at consultations was also compared; 36 (69.2%) patients maintained regular follow-up, with the majority in the group that showed clinical/immunological/virological improvement, although without statistical significance. In the group of patients who improved, 30 (57.7%) had regular attendance compared to 14 (26.9%) who had irregular attendance at consultations (p=1.00).

Of the total patients, 34 (65.4%) had records of viral genotyping tests, allowing for the identification of the viral subtype. Among these, 19 (55.9%) were subtype C, 11 (32.3%) were subtype B, 2 (5.9%) were subtype F1, and 2 (5.9%) had no identification. Of these, 14 (41.2%) exhibited some form of resistance, with 3 (21.4%) categorized as primary genotyping resistance, while the remaining were tests conducted for therapeutic failure evaluation. In the primary resistance cases, all demonstrated only resistance to nucleoside reverse transcriptase inhibitors (NRTIs). The overall resistance profile was: 9 (81.8%) resistant to non-nucleoside reverse transcriptase inhibitors (NNRTI), 4 (36.4%) resistant to Integrase Strand Transfer Inhibitor (INSTI), 3 (27.3%) resistant to NRTI, 1 (2.1%) resistant to protease inhibitor (PI), and 1 (2.1%) of indeterminate resistance.

In the administration of ART for these patients, 45 (86.6%) were using current first-line drugs, 5 (9.6%) were on therapeutic failure regimens, 1 (1.9%) was on an old first-line regimen, and 1 (1.9%) was on a simplified regimen due to having an undetectable viral load and an adverse event related to the medication. The average need for regimen change was 2.8 switches (±1.44). The current ART regimen, by class, is described in Table 4. Dolutegravir, which is a more recent first choice as integrase inhibitor (INI) in first-line drug therapy, was present in 32 (71.1%) of the first-line regimen users. Furthermore, among all patients with genotyping, only one presented resistance to dolutegravir.

**Table 4:** Frequency of antiretroviral therapy use, by treatment regimen, in children and adolescents living with human immunodeficiency virus, seen at a reference service in Southern Brazil, 2022.

Class of ART	n	%
2 NRTIs + 1 INI	36	69.3
2 NRTIs + 1 PI	9	17.3
2 NRTIs + 1 PI + 1 INI	4	7.7
2 NRTIs + 1 NNRTI + 1 PI	1	1.9
2 NRTIs + 1 NNRTI	1	1.9
1 PI + 1 INI	1	1.9

ART: antiretroviral therapy; NRTI: nucleoside reverse transcriptase inhibitor; INI: integrase inhibitor; NNRTI: non-nucleoside reverse transcriptase inhibitor; PI: protease inhibitor.

## DISCUSSION

The number of infections due to vertical transmission has been declining worldwide in recent years.^10^ In 1989, 25% of children born to HIV-positive mothers were infected by the age of 2, whereas today, in wealthy countries, those figures have dropped to 1–2%.^11^ Currently, vertical transmission through breastfeeding accounts for about 50% of pediatric HIV infections worldwide.^12^ In our population, transmission through breastfeeding has remained stable, while there has been a decline in intrauterine or intrapartum transmission. A concerning statistic is the high median age of diagnosis for these patients, reflecting a failure in maternal diagnosis during prenatal care or a transmission that occurs during breastfeeding. Therefore, an important discussion is the need for periodic maternal retesting while breastfeeding. It is known that HIV in children can behave as a slow progression, taking years to manifest,^5,6^ and thus diagnosis through screening tests can improve the outcomes for these children.

Another point is the low latent tuberculosis screening in these patients, which is recommended annually. It is a sensitive point, as it is a coinfection that may interfere with the evolution of the HIV infection and alter the chosen ART.^5,6^ The low screening is associated with disponible problems of the Purified Protein Derivative (PPD) used and the logistics involved in its usage (necessity of return on another date to get the result), a situation that may be improved with the crescent disponible of the Interferon-Gamma Release Assay (IGRA) test.

The current guidelines for children and adolescents living with HIV/AIDS^5,6^ indicate that the first-choice therapy among this population is a combination of three drugs — two NRTIs + 1 INI or 1 PI. The use of NNRTIs is no longer routinely recommended. Being so, our population is in accordance with the current guidelines.

Our rate of resistance to any class of antiretrovirals was still above the 33% found in another study of a population similar to ours.^13^ In both studies, the most common type of resistance was to NNRTIs. Although our population showed worse results regarding resistance to INIs, the resistance to PIs was similar.^13,14^ The presence and profile of resistance are related to various factors, such as access to medication, palatability, and drug interactions, among others. Viral subtypes are also a factor to consider regarding the presence of resistance mutations, with subtype B being 2.2 times more likely to develop resistance.^14^ Regarding the resistance found in the primary genotypings analyzed, our prevalence was higher than the 10% observed in other Western populations.^13^ However, once again, the most common resistance was to NNRTIs. The presence of transmitted resistance highlights the importance of the mother-child relationship in controlling pediatric HIV, making it essential to also care for women of childbearing age to improve the care of this population.^15^

The most relevant finding in this study was that our population improved (clinically and laboratory) with the current follow-up. This supports the assertion that the use of ART reduces blood viral load while also improving CD4 levels.^15^ Thus, the medical follow-up of these children is essential for the effective management of the disease and ART must always be guaranteed. However, despite the pediatric population being a challenging group to retain for follow-up,^1^ one point of interest emerged: in our analysis, no statistical difference was observed when comparing improvement or worsening of the condition with regular or irregular follow-up. Since irregular follow-up was defined as missed appointments, this may not accurately reflect the patient’s actual adherence to the therapeutic plan. The historic evolution of treatment recommendations also takes in consideration the factors to improve adherence, such as palatability and less daily intakes,^5,6^ and the recent switch to dolutegravir may be a weapon to combat the abandonment of treatment. To better understand the reasons behind clinical and laboratory improvement following the start of care in a specialized service, even with missed appointments, new studies focused on this specific analysis are necessary. Other parameters that are usually associated with better outcomings, age and duration of service,^16,17^ did not show statistically significant relevance for determining treatment success in our population.

Finally, our population still has a higher rate of patients with undetectable viral load than the 36% reported worldwide in a study from 2021,^18^ but we are still far from the 90%^2^ target. Therefore, we need a better therapeutic arsenal, including different drugs with improved formulation and palatability, as well as more data to better understand our patients’ adherence to treatment and its consequences for viral suppression and clinical/laboratory outcomes.

These data can inform public health policies for quality follow-up, reducing long-term harmful effects. HIV infection in children remains a persistent problem and can lead to fatal complications.

The main limitation of our study is that it is based on the analysis of medical records over a short period of time. Additionally, it is subject to recording errors or missing information.

In conclusion, children followed at the Pediatric Infectology Service of CHC-UFPR showed improvements in viral load levels, immunosuppression, and clinical status. However, the rate of patients with undetectable viral load is still far from the 90% target set by Joint United Nations Programme on HIV/AIDS (UNAIDS). It is necessary to better understand, through more qualitative studies, the reasons why children are not improving as much as they could. Factors include social risk, medication tolerability, access to services and treatment, among many others.

## Data Availability

The database that originated the article is available, with the corresponding author.
